# Supplementary material for: Hotspots for social and ecological impacts from freshwater stress and storage loss
Source: Nat Commun. 2022 Jan 21;13:439. doi: 10.1038/s41467-022-28029-w (PMC8783008; doi:10.1038/s41467-022-28029-w)
Supplement: Supplementary file 1 — supplementary information [file 41467_2022_28029_MOESM1_ESM.pdf]

Supplementary Information

Hotspots for social and ecological impacts from freshwater stress and storage loss

Xander Huggins[1,2], Tom Gleeson[1,3]*, Matti Kummu[4], Samuel C Zipper[5], Yoshihide Wada[6], Tara J. Troy[1], James S. Famiglietti[2,7,8]
[1] Department of Civil Engineering, University of Victoria, Canada
[2] Global Institute for Water Security, University of Saskatchewan, Canada
[3] School of Earth and Ocean Sciences, University of Victoria, Canada
[4] Water and Development Research Group, Aalto University, Finland
[5] Kansas Geological Survey, University of Kansas, USA
[6] International Institute for Applied Systems Analysis, Austria
[7] School of Environment and Sustainability, University of Saskatchewan, Canada
[8] Department of Geography and Planning, University of Saskatchewan, Canada

Table of Contents:

Section 1: Overview of general study approach **2**
Supplementary Fig. 1: Conceptual model of the study. 3
Supplementary Fig. 2: Zones of the conceptual model for individual description. 3
Section 2: Data selection and preprocessing **7**
Supplementary Table 1: Data sources, description, justification, and summary of any data preprocessing applied. 7
Section 3: Supplementary methodology figures **12**
Supplementary Fig. 3: Overview schematic of the hotspot derivation process. 12
Supplementary Fig. 4: Data inputs and derivation steps of basin freshwater status. 13
Supplementary Fig. 5: Data inputs and derivation of the ecological sensitivity indicator. 15
Supplementary Fig. 6: Adaptive capacity. 16
Supplementary Fig. 7: Derivation of the social-ecological sensitivity indicator. 16
Section 4: Uncertainty and sensitivity analysis methods and results **17**
Supplementary Fig. 8: Analysis of potential spatially uniform data uncertainty. 20
Supplementary Fig. 9: Analysis of potential spatially variable data uncertainty. 21
Supplementary Fig. 10: The sensitivity of hotspot basin results to subjective aspects of this study's methodology. 22
Section 5: Supplementary results **23**
Supplementary Table 2: Wetlands of international importance (Ramsar Sites) located in hotspot basins. 23
References **23**

Section 1: Overview of general study approach

Conceptualizing and quantifying interactions between humans, ecosystems, and hydrological processes are critical to broad sustainability efforts across all scales[1–3]. To date, however, very few studies consider the complex interplay between humans, ecosystems, and hydrology at the global scale. To our knowledge, only one global study (Varis et al.[4]) integrates social, ecological, and hydrological considerations in a broad resilience framework. While the study of Varis et al. is highly relevant, and we implement their social adaptive capacity dataset in our study, our approach is specific to the potential impacts from co-occurring freshwater stress and storage loss on social and ecological systems. In contrast, Varis et al. evaluate the general ability of social adaptive capacity to offset broadly defined ecological vulnerability.

Motivated by the conceptual template of biodiversity hotspots[5], we sought to apply social-ecological system principles to identify the basins most vulnerable to freshwater stress and freshwater storage loss in hopes of steering policy agendas and scientific focus to the identified basins. As process-based knowledge of human-water system interactions at the global scale is characterized by deep uncertainty, we performed a parsimonious, spatial analysis to quantify basin vulnerability to freshwater stress and storage loss based on general social-ecological system principles.

We provide a conceptual model of our study in Supplementary Figure 1, which visualizes the relationships of all elements considered in our methodology and discussion. These relationships are not intended to be exhaustive; rather, they are shown to clarify the core conceptual underpinnings of our study.

Noting the intertwined relationships between vulnerability, resilience, and adaptive capacity[6], we find it important to clarify our terminology and specifically why we describe this study as a vulnerability analysis. Whereas vulnerability and adaptive capacity can be generally considered as system states, resilience is a more dynamic property with an inherent temporal dimension. More simply, resilience and vulnerability may be best approached through dynamic and static frames of analysis, respectively, and are linked through the concept of adaptability[6]. As global social-ecological data is not readily available at sufficient temporal resolution or for particular processes that would enable resilience considerations to be addressed, we performed a static vulnerability analysis. Resilience concepts were invoked in our discussion, however, through the feedback mechanisms shown in the conceptual model.

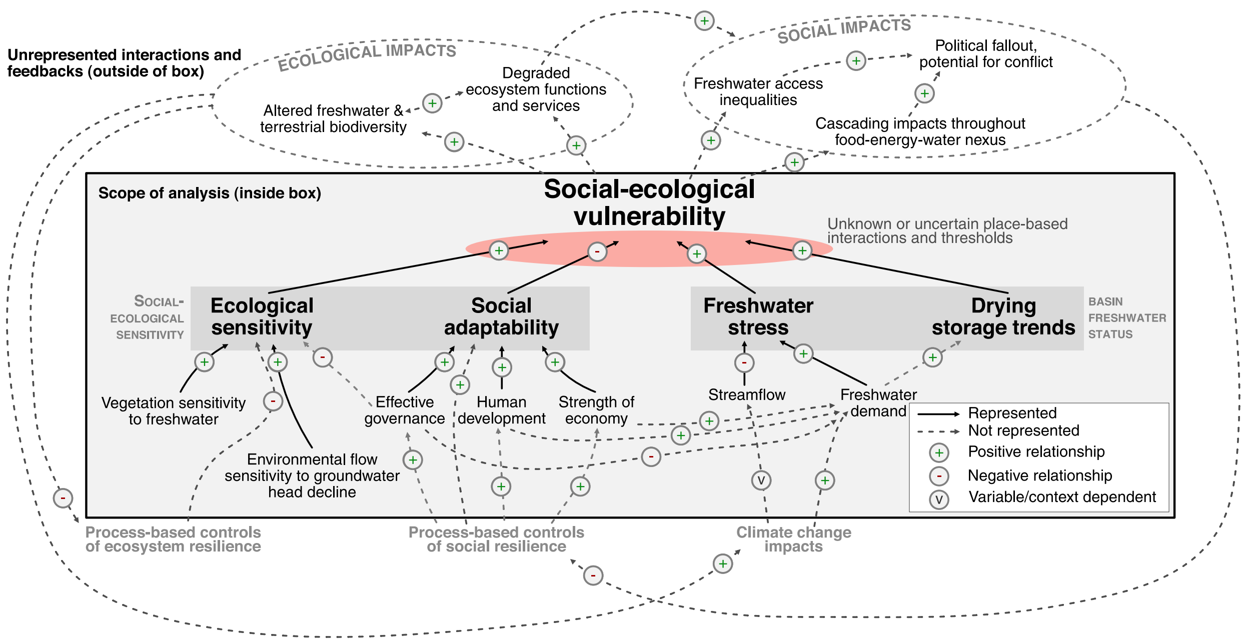

Supplementary Fig. 1: Conceptual model of the study. Core variables and concepts incorporated in this study and our interpretation of the relationships between them. Elements represented in our analysis are shown by solid lines and are found within the black-outlined box (denoted by the 'scope of analysis' label). Elements and relationships that were not represented in our analysis but were discussed in the main text are found outside of this box and/or are shown by dashed lines. Positive (negative) relationships between two variables (first variable → second variable) represent relationships where we consider an increase in the first variable to lead to an increase (decrease) in the second variable.

We dedicate the remainder of this section to providing addition details on the conceptual model in a zonal approach as specified by Supplementary Figure 2.

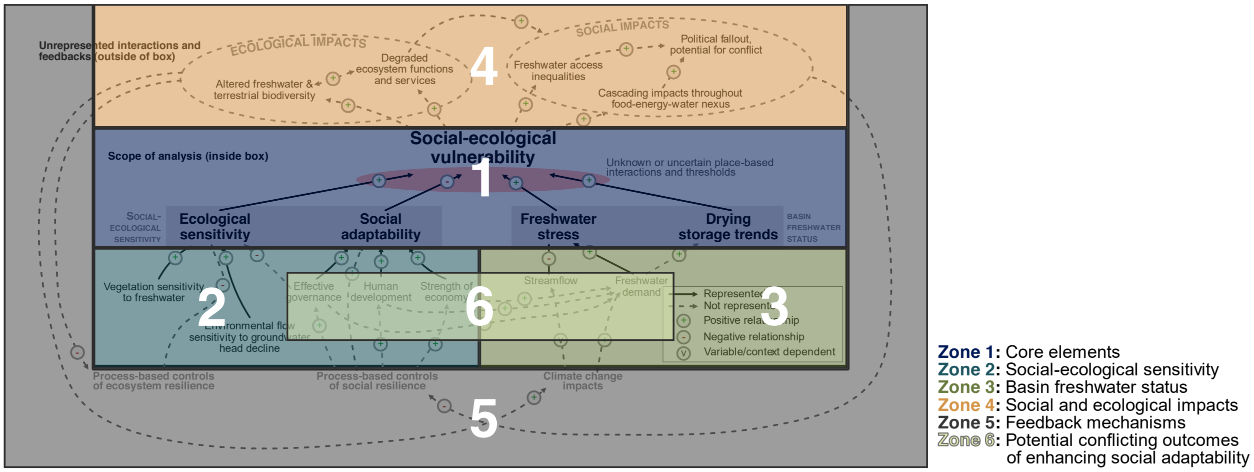

Supplementary Fig. 2: Zones of the conceptual model receiving supplemental description, individually.

Zone 1: Core elements

Conceptually, we orient our vulnerability analysis around the broad definition of Turner et al.[7] where "vulnerability is the degree to which a system … is likely to experience harm due to exposure to a hazard, either a perturbation or stress/stressor", which can be represented by the combined consideration of exposure to a hazard, system sensitivity, and adaptive capacity[6]. The concept of sensitivity has many potential meanings in this study: it can imply the sensitivity of flow regimes to changes in freshwater storage, the sensitivity of ecosystems to changes in streamflow or in freshwater storage, or the sensitivity of human activities and well-being to hydrological perturbations. While we do not consider this first interpretation of sensitivity, it can be partially inferred through our combination of freshwater stress and storage trends. We do consider the latter two interpretations, which we refer to together as social-ecological sensitivity. Thus, we interpret sensitivity and adaptive capacity concepts from ecohydrological and sociohydrological perspectives, respectively, and not from a strictly hydrological perspective. (Note that this discussion on system sensitivity should not be confused with our sensitivity analysis in Supplementary Section 4.)

Guiding our governing equation (equation 1 in Methods), we considered social-ecological vulnerability to be a function of basin freshwater status (see Zone 3 description) and social-ecological sensitivity. While the input indicators for basin freshwater status and social-ecological sensitivity (i.e. freshwater stress, storage loss, ecological sensitivity, and social adaptability) all affect social-ecological vulnerability, specific threshold relationships between these four variables have yet to be identified or sufficiently studied.

Zone 2: Social-ecological sensitivity

In our derivation of ecological sensitivity, we combined two global datasets that consider separate facets of ecological sensitivity to freshwater stress and storage trends: (1) vegetation sensitivity to anomalies in soil moisture and shallow groundwater storage, and (2) environmental flow sensitivity to simulated groundwater head decline. There are many other processes that contribute to ecosystem-wide sensitivity to freshwater stress and storage trends, though we are not aware of any others that have been considered in global analyses with accompanying open data products. For instance, there remains no global mapping of groundwater dependent ecosystems, or global analyses of place-based aquatic and terrestrial biodiversity sensitivity to environmental flow transgressions.

Our representation of social adaptive capacity was derived by Varis et al.[4], who understood social adaptive capacity to represent the broad ability of the "social part of social-ecological systems" to both reactively and proactively adapt and increase resilience to ecological vulnerabilities. Varis et al. derived adaptive capacity from three input indicators: government effectiveness, strength of economy, and human development. We note that this is a relatively parsimonious conceptualization of adaptive capacity which may be challenged to represent critical dynamic properties of the social system, such as its absorbability or transformability[8].

Zone 3: Basin freshwater status

We derived the basin freshwater status indicator to compress the bivariate relationship between freshwater stress and freshwater storage trends into a single dimension, which we

discuss and describe in the main text and Methods. As freshwater demand is attributed as a driver of large-scale drying in several mid-latitude regions globally[9], we link freshwater demand to drying trends in the conceptual model. However, as the storage trend data we used were derived from satellite observations (see Supplementary Table 1) and not estimated from hydrological model with human activity representations, this relationship is shown by a dashed (i.e. unrepresented) line in the conceptual model.

Zone 4: Social and ecological impacts
Despite the aforementioned knowledge gap on processes and thresholds of large-scale social-ecological systems in the context of freshwater stress and storage loss, our conceptual model assumes that increases in our derived indicator of social-ecological vulnerability leads to a greater likelihood that social and ecological impacts are experienced within the system. All ecological and social impacts shown in the conceptual model are discussed in the main text.

Zone 5: Feedback mechanisms
Resilience is represented by the feedback mechanisms shown in the conceptual model that act on the underlying process-based controls of ecosystem and social functions. Ecological impacts, as identified in Zone 4, reduce ecosystem resilience by decreasing system diversity, connectivity, and altering 'slow variables' that regulate ecosystem functioning[10]. Ecological impacts can also feed back to affect freshwater stress and storage trends through altered ecosystem services , climate dynamics, and their interactions. Where regulating ecosystem services are degraded, the impacts of climate change may be exacerbated which may result in increasing rates of storage loss, increasing rates of freshwater demand, and decreasing or more-variable streamflow rates[11]. Social impacts of freshwater stress and storage loss can feed back and deteriorate process-based controls of social resilience, though we note that literature is sparse on cascading impacts of hydrological hazards in large-scale social-ecological systems.

Zone 6: Potential for conflicting outcomes when enhancing social adaptability
Considering the unique role of humans in driving the vulnerability of the social-ecological system, we identify a certain tension between the input variables used to derive social adaptive capacity and their impact on freshwater stress and storage trends. For example, human development and economic strength are identified as positive contributors to social adaptive capacity yet both are commonly associated with increases in freshwater demand that drive freshwater stress and storage loss. Thus, human development and economic growth may produce an offsetting effect on social-ecological vulnerability by simultaneously increasing freshwater stress and storage loss. There exists, therefore, a need for effective governance to balance these impacts, such as through linking Sustainable Development Goals, as we discuss in the main text.

Limitations of the conceptual model and approach
Several important processes and considerations are absent from our conceptual model. These include the absence of global trade considerations, or the role of cross-basin water transfer infrastructure projects. We also performed a 'lumped' analysis of social-ecological vulnerability

at the basin scale and thus sub-basin variability of all input variables was not considered. Thus, basins with particularly large variance in input parameters may be poorly represented by the basin average.

Section 2: Data selection and preprocessing

All data used in this study are described and justified in Supplementary Table 1.

Supplementary Table 1: Data sources, description, justification, and summary of any data preprocessing applied.

**Dataset**	**Data Source.**<br>**Persistent Web Link.**<br>**Temporal Range.**<br>**Spatial Resolution.**<br>**Resolution Harmonization Method.**<br>**Description and Justification.**<br>**Additional Preprocessing.**
**Freshwater withdrawal and consumption rates**	**Data Source:** Huang et al.[12,13]<br>**Persistent Web Link:**<br>https://doi.org/10.5281/zenodo.1209296<br>**Temporal Range:** 1971 – 2010, monthly.<br>**Spatial Resolution:** 0.5°<br>**Resolution Harmonization Method:** N/A<br>**Description and Justification:** Monthly withdrawal and consumption rates for six water use sectors: irrigation, domestic, electricity generation, livestock, mining, and manufacturing. Irrigation estimates are provided from four hydrological models: WaterGAP, H08, LPJmL, and PCR-GLOBWB. Non-irrigation sector estimates are spatially downscaled based on global population and livestock density maps.<br>**Additional Preprocessing:** As this dataset was not available for our desired year of 2015, we aggregated all sectors over the most recent year available, 2010. To reconcile the four alternative irrigation estimates, we calculated the median of the four model alternatives at the individual grid cell and used these rates in addition to the other five sectors in our representation of total freshwater withdrawal and consumption rates. We used withdrawal rates data in our main analyses, but considered the impact of this decision by also deriving hotspot basins using consumption rates in our sensitivity analysis (Supplementary Section 4).
**Streamflow**	**Data Source:** Global Streamflow Characteristics Dataset[14-16] and Ghiggi et al. (GRUN)[17,18]<br>**Persistent Web Link:** http://www.gloh2o.org/gscd/ and https://doi.org/10.6084/m9.figshare.9228176<br>**Temporal Range:** N/A (reference) and 1902-2014, respectively<br>**Spatial Resolution:** 0.5°<br>**Resolution Harmonization Method:** N/A<br>**Description and Justification:** The Global Streamflow Characteristics Dataset (GSCD) provides spatially-distributed, global maps of 17

	streamflow characteristics derived from neural network ensembles trained with observed streamflow records, including mean annual flow (Q_{mean}). The Q_{mean} hydrological signature was trained over the record of available streamflow observations and does not correspond directly with a calendar year. GRUN is an alternate global, gridded streamflow dataset derived through a neural network trained with streamflow observations. Conversely to GSCD, GRUN reconstructs historical runoff at monthly time steps over 1902-2014. We used the GSCD dataset in our main analyses based on its more-frequent use as a reference dataset, and we considered the impact of this decision in our sensitivity analysis.<br>**Additional Preprocessing:** We estimated Q_{mean} from GRUN, for use in the sensitivity analysis, by calculating average annual streamflow over 2000-2010.
**Freshwater storage trends**	**Data Source:** Rodell et al.[9]<br>**Persistent Web Link:** https://doi.org/10.1038/s41586-018-0123-1<br>**Temporal Range:** April 2002 – March 2016.<br>**Spatial Resolution:** 0.5°<br>**Resolution Harmonization Method:** N/A<br>**Description and Justification:** Preprocessed annual trends in terrestrial water storage (TWS) from the Gravity Recovery and Climate Experiment (GRACE) satellite mission. TWS trends represent annual trends in combined groundwater, soil moisture, surface water, canopy water, ice and snow storages. GRACE observations are the only observation-based dataset of trends in global freshwater storage. While the ongoing GRACE Follow-On (GRACE-FO) Mission provides data to update the GRACE trends to present, they are not used in our study as they exceed our target year of 2015.<br>**Additional Preprocessing:** N/A
**Vegetation sensitivity**	**Data Source:** Seddon et al.[19,20]<br>**Persistent Web Link:** https://doi.org/10.5287/bodleian:VY2PeyGX4<br>**Temporal Range:** 2000-2013<br>**Spatial Resolution:** 0.05°<br>**Resolution Harmonization Method:** Average resampling to 0.5° (arithmetic mean of all contributing raw pixels, n = 100)<br>**Description and Justification:** Seddon et al. derived a vegetation sensitivity index (VSI) to identify ecosystem sensitivity to three climate variables: air temperature, water availability, and cloud cover. The VSI is derived from a seasonally detrended time series of the enhanced vegetation index (EVI) and the three climate variables. The VSI is calculated as the log_{10}-transformed ratio of EVI to climate variance weighted based on the relative importance of each climate variable. The ratio of actual evapotranspiration to potential evapotranspiration is used as the proxy indicator of water availability. As the VSI is a composite

	indicator across the three climate variables, we used only the contribution of water availability to the overall VSI.<br>**Additional Preprocessing:** N/A
**Environmental flow sensitivity**	**Data Source:** de Graaf et al.[21,22]<br>**Persistent Web Link:** https://doi.org/10.5683/SP2/D7I7CC<br>**Temporal Range:** 1960-2100<br>**Spatial Resolution:** 0.0833° (5 arc-minute)<br>**Resolution Harmonization Method:** Average resampling to 0.5° (arithmetic mean of all contributing raw pixels, n = 36)<br>**Description and Justification:** de Graaf et al. modelled the groundwater head decline at which a presumptive environmental flow limit is transgressed for all basins where groundwater pumping occurs. The model used in the study was PCR-GLOBWB coupled to a two-layer groundwater model that simulated lateral flow[13]. To date, no other global study has quantified the sensitivity of environmental flow transgression to groundwater pumping.<br>**Additional Preprocessing:** N/A
**Social adaptive capacity**	**Data Source:** Varis et al. [4,23]<br>**Persistent Web Link:** https://doi.org/10.5061/dryad.h2v2398<br>**Temporal Range:** 2015<br>**Spatial Resolution:** 0.0833° (5 arc-minute)<br>**Resolution Harmonization Method:** Average resampling to 0.5° (arithmetic mean of all contributing raw pixels, n = 36)<br>**Description and Justification:** Varis et al. derived an indicator of social adaptive capacity through an equal-weighted composite of governance, economic (GDP per capita) and human development indicators. To our knowledge, no other spatially distributed dataset exists that represents the general capacity of the social system to respond to broad environmental disturbances.<br>**Additional Preprocessing:** N/A
**Population count**	**Data Source:** Gridded Population of the World – United Nations World Population Prospects version 4 (GPW UN-WPP Adjusted v4.11) [24,25]<br>**Persistent Web Link:** https://doi.org/10.7927/H4PN93PB<br>**Temporal Range:** 2015<br>**Spatial Resolution:** 0.5°<br>**Resolution Harmonization Method:** N/A<br>**Description and Justification:** Global gridded population count adjusted to match UN-WPP country totals. This dataset is the recommended GPW dataset for global analyses. This dataset is interchangeable with the Global Human Settlement Population (GHS-POP) dataset at coarse resolutions (i.e. 0.5°) as GHS-POP is a further downscaled product of GPW.<br>**Additional Preprocessing:** N/A

**Food crop production**	**Data Source:** Kummu et al.[26,27]<br>**Persistent Web Link:** N/A see code repository associated with above reference: https://github.com/matheino/holdridge<br>**Temporal Range:** 2010<br>**Spatial Resolution:** 0.0833° (5 arc-minute)<br>**Resolution Harmonization Method:** Aggregated (summed) to 0.5°<br>**Description and Justification:** Kummu et al. converted crop production data from the SPAM dataset[25] into combined kilocalories for 27 major food crops for the year 2010. While more recent gridded agricultural products exist, none have been converted and combined into total kilocalorie production and would require additional processing. Thus, the Kummu et al. dataset represents the most ready-for-use dataset for our purposes, despite not being aligned with our desired year of 2015.<br>**Additional Preprocessing:** N/A
**Gross Domestic Product**	**Data Source:** Kummu et al.[28,29]<br>**Persistent Web Link:** https://doi.org/10.5061/dryad.dk1j0<br>**Temporal Range:** 2015<br>**Spatial Resolution:** 0.00833° (30 arc-second)<br>**Resolution Harmonization Method:** Aggregated (summed) to 0.5°<br>**Description and Justification:** Gross domestic product (GDP) in 2011 international USD. While an alternative UNEP/GRID Geneva gridded 2010 GDP dataset exists, Kummu et al.'s alternative is more aligned with FAIR (findable, accessible, interoperable, and reusable) data principles as well as with our desired year of 2015.<br>**Additional Preprocessing:** N/A
**Amphibian species richness**	**Data Source:** IUCN amphibian richness grids[30]<br>**Persistent Web Link:** https://doi.org/10.7927/H4RR1W66<br>**Temporal Range:** 2015<br>**Spatial Resolution:** 0.00833° (30 arc-second)<br>**Resolution Harmonization Method:** Maximum resampling to 0.5° (maximum of contributing raw pixels, n = 60)<br>**Description and Justification:** The number of amphibian species present per grid cell. We opted for these richness grids rather than the Global Freshwater Biodiversity Atlas as they are globally available in distributed grid format rather than at the basin scale with sub-global coverage as is provided by the Biodiversity Atlas. We selected amphibian species richness grids rather than other taxa following Tisseuil et al.[31] who recommend that amphibians be used as a surrogate candidate for global freshwater conservation planning.<br>**Additional Preprocessing:** N/A
**Priority wetlands for conservation**	**Data Source:** Ramsar List of wetlands of international importance[32]<br>**Persistent Web Link:** https://rsis.ramsar.org/<br>**Temporal Range:** Last accessed 20 March 2021

	**Spatial Resolution:** Point data<br>**Resolution Harmonization Method:** N/A<br>**Description and Justification:** A list of wetlands of international importance "on account of their international significance in terms of ecology, botany, zoology, limnology, or hydrology"[30] which must satisfy at least one of nine criteria. There are currently over 2,400 Ramsar Sites.<br>**Additional Preprocessing:** N/A
**Integrated water resources management implementation**	**Data Source:** IWRM Data Portal[33]<br>**Persistent Web Link:** http://iwrmdataportal.unepdhi.org/countrydatabase<br>**Temporal Range:** 2017, 2020<br>**Spatial Resolution:** National boundaries<br>**Resolution Harmonization Method:** Minimum value polygon rasterization to 0.5°<br>**Description and Justification:** The IWRM Data Portal records and presents "the global status and progress on SDG 6.5.1", which is "[the] degree of integrated water resources implementation". The Data Portal tracks SDG 6.5.1 progress through 33 indicators, across four IWRM components: an enabling environment, institutions and participation, management instruments, and financing. Baseline data is provided for the year 2017 and an updated dataset is provided for the year 2020.<br>**Additional Preprocessing:** We primarily used the more recent (2020) dataset. However, we filled any gaps in the 2020 dataset if nations received IWRM scores in 2017 but did not participate in the 2020 update.
**Basins**	**Data Source:** HydroBASINS[34,35]<br>**Persistent Web Link:** https://www.hydrosheds.org/page/hydrobasins<br>**Temporal Resolution:** N/A<br>**Spatial Resolution:** 15-arcseconds<br>**Resolution Harmonization Method:** Borders simplified to a 0.5° grid.<br>**Description and Justification:** HydroBASINS are the global standard in basin discretization schemes. We used HydroBASINS level 4 in our main analyses, however we considered the impact of this decision by including levels 3 and 5 in our sensitivity analysis.<br>**Additional Preprocessing:** N/A

Section 3: Supplementary methodology figures

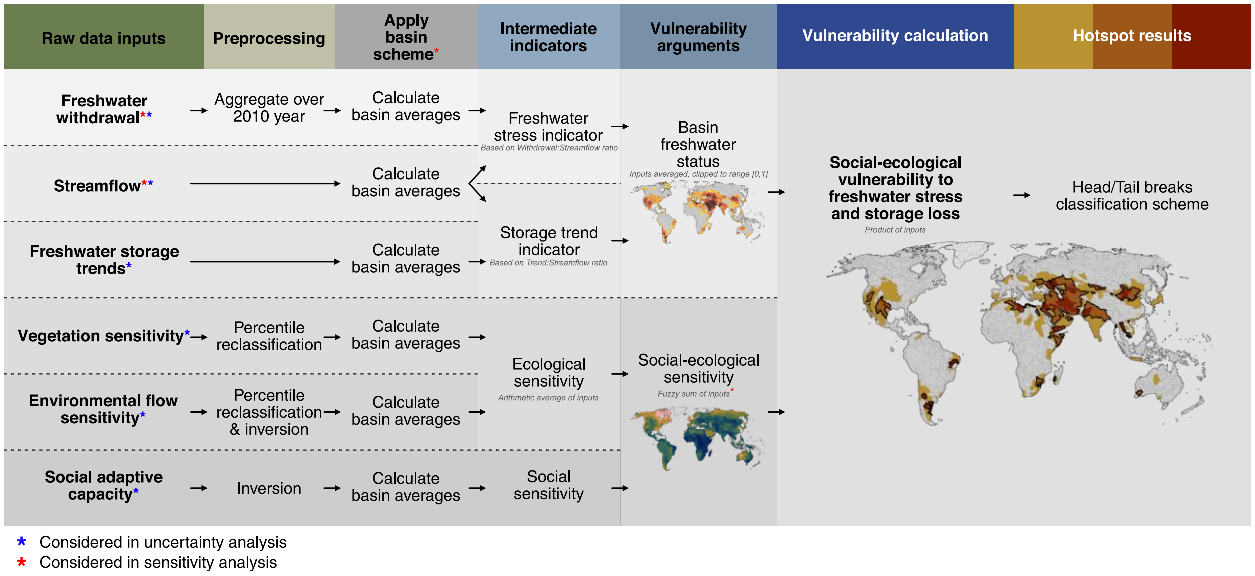

Supplementary Fig. 3: Overview schematic of the hotspot basin derivation process. To be viewed from left to right: (i) raw data inputs were preprocessed if necessary, then (ii) these inputs were summarized to the selected basin scheme, (iii) combined into intermediate indicators, and subsequently (iv) combined into vulnerability function arguments. The final vulnerability dataset, a product of both vulnerability arguments, was classified into vulnerability classes and hotspot basins using the Head/Tail breaks classification scheme. Data resolution harmonization methods are listed in Supplementary Table 1 and are not shown in the 'preprocessing' column. Blue asterisks indicate the variables we considered in our uncertainty analysis (Supplementary Fig. 8,9). Red asterisks indicate the methodological elements we considered in our sensitivity analysis (Supplementary Fig. 10).

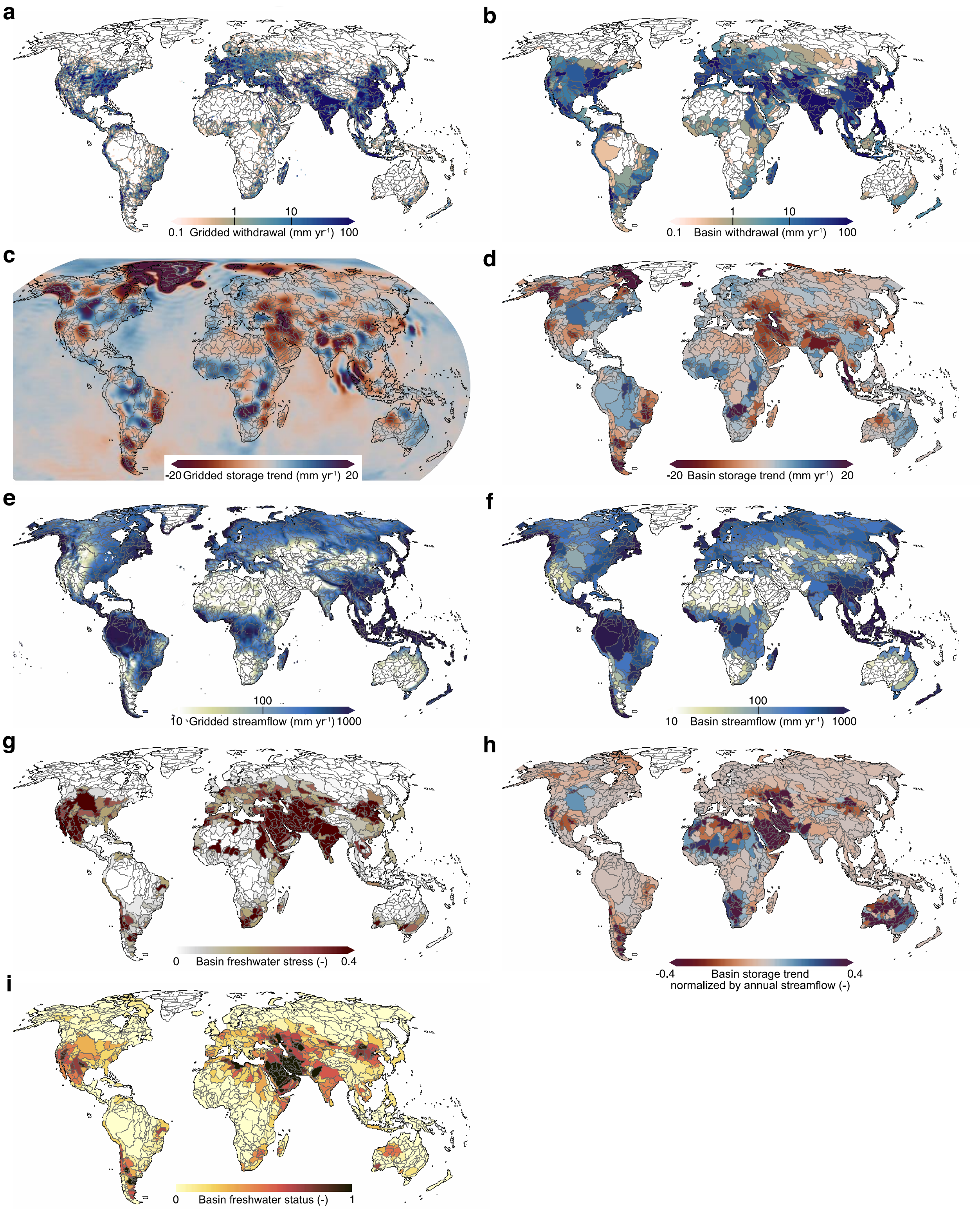

Supplementary Fig. 4: Data inputs and derivation steps of basin freshwater status. **a**, Gridded annual freshwater withdrawals. **b**, Average annual freshwater withdrawals per basin. **c**, Gridded trends in freshwater storage. **d**, Trends in freshwater storage per basin. **e**, Gridded annual streamflow. **f**, Annual streamflow per basin. **g**, Basin freshwater stress, calculated as

withdrawals divided by streamflow. **h**, Basin freshwater storage trend normalized by streamflow, calculated as storage trends divided by streamflow. **i**, Basin freshwater status, derived from normalized inputs of panels **g** and **h**, as described in the Methods.

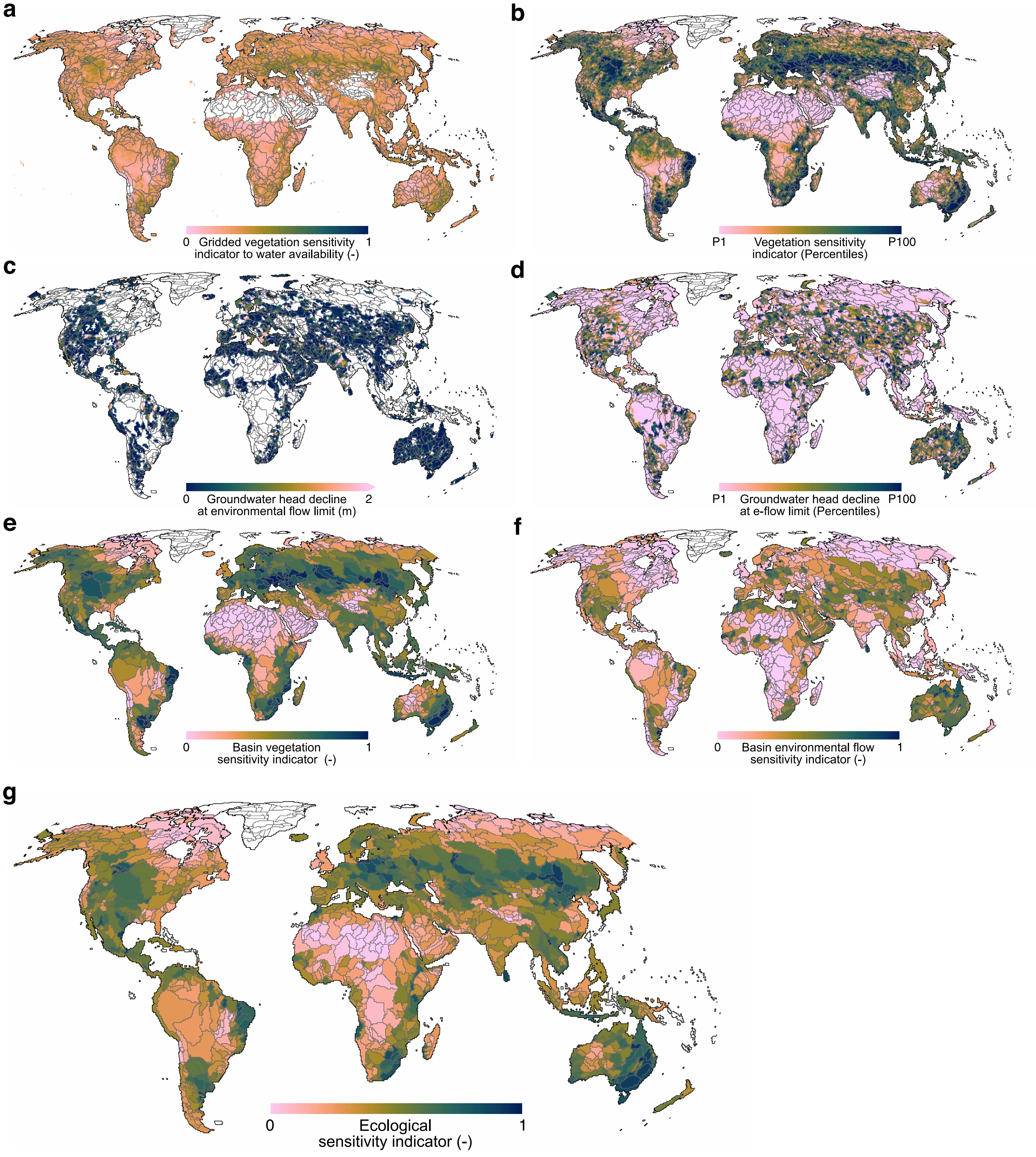

Supplementary Fig. 5: Data inputs and derivation of the ecological sensitivity indicator. a, Vegetation sensitivity to water anomalies from the Vegetation Sensitivity Index[17]. **b**, Percentile reclassification of panel a. **c**, Groundwater head decline at which environmental flow limits are transgressed[19]. **d**, Inverted percentile reclassification of panel c. This dataset was inverted as threshold transgressions at smaller head declines represent greater sensitivity. **e**, Basin average vegetation sensitivity percentiles (shown in b). **f**, Basin average environmental flow sensitivity percentiles (shown in d). **g**, Combined ecological sensitivity indicator, calculated as the average of maps e and f, and normalised by the maximum basin value.

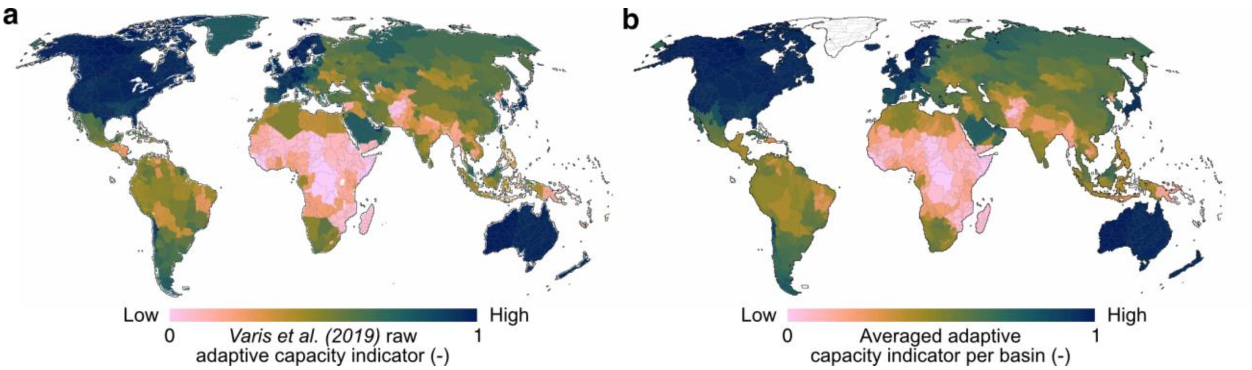

Supplementary Fig. 6: Adaptive capacity. a, Social adaptive capacity, as derived by Varis et al[4]. **b**, Social adaptive capacity per basin, as used in our analysis.

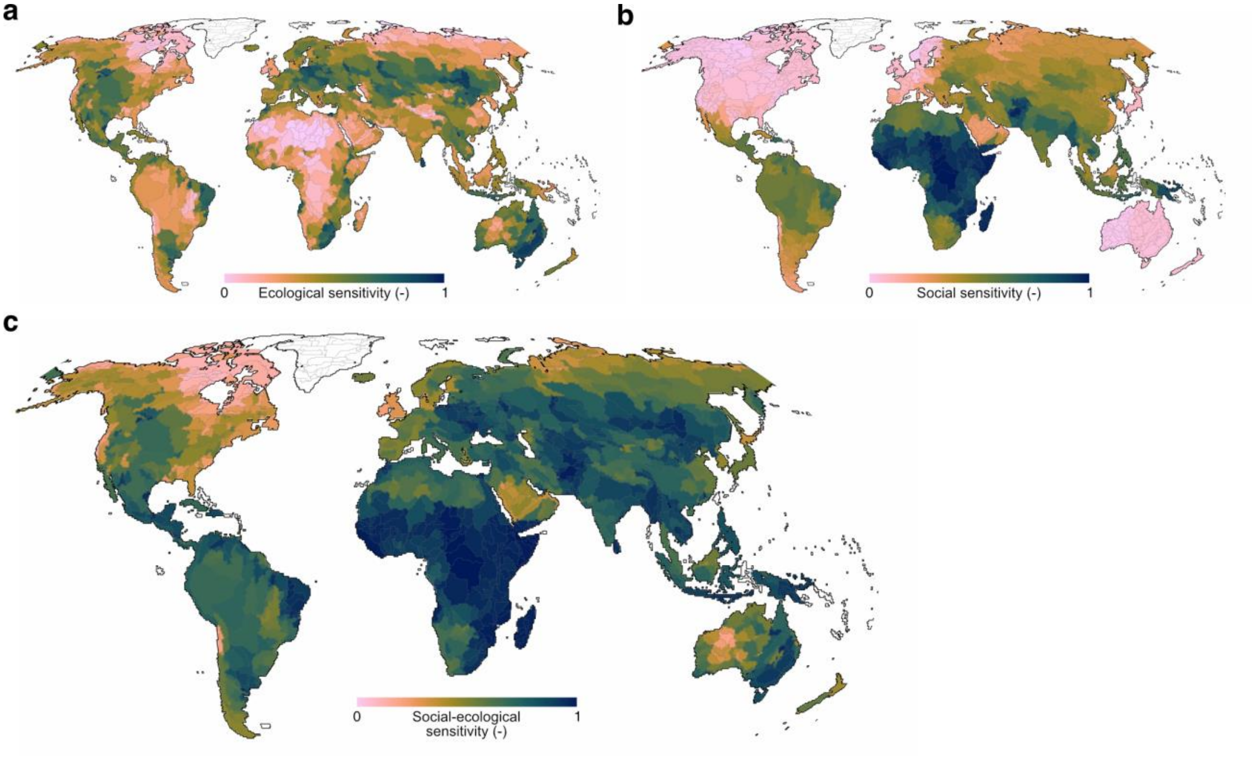

Supplementary Fig. 7: Derivation of the social-ecological sensitivity indicator. a, Ecological sensitivity indicator (as shown in Fig. 5g). **b**, Social sensitivity indicator (inverted from Supplementary Fig. 6b). **c**, Social-ecological sensitivity, calculated as the fuzzy sum of a and b.

Section 4: Uncertainty and sensitivity analysis methods and results

As our multidimensional input datasets possess either spatially variable or unquantified uncertainty, we opted for a parsimonious perturbation-based approach to explore the impact of input data uncertainty on hotspot basin results. We considered two forms of data uncertainty: systematic under- or over-estimation of individual parameters (hereafter referred to as spatially uniform uncertainty), and spatially variable uncertainty. We also conducted a separate sensitivity analysis that explores the impact of subjective decisions made in our methodology on our hotspot results.

To explore potential spatially uniform uncertainty, we simultaneously varied the basin-level input data for all six input variables to our hotspot analysis: freshwater withdrawal rates (mm yr^{-1}), streamflow (mm yr^{-1}), storage trends (mm yr^{-1}), social adaptability (-), percentile-reclassified vegetation sensitivity (-), and percentile-reclassified environmental flow sensitivity (-). For each perturbation set, all basins are perturbed by the same magnitude for each variable. The perturbations between variables and between sets were uncorrelated, where the perturbation applied per variable per set was randomly sampled from a normal distribution $N(\mu = 0, \sigma = 10\%$ of the global σ of the variable). We repeated the hotspot analysis for 10,000 perturbation sets. Supplementary Figure 8 reveals the results of our analysis of potential spatially uniform uncertainty. We found that the number of identified hotspot basins increased as withdrawal rates increased, and storage trends became more negative. We found interesting patterns in streamflow perturbations, where a bimodal distribution was found for negative perturbations while no clear effect was identified for positive perturbations. No trend was visible in perturbations applied to social adaptive capacity, vegetation sensitivity, or environmental flow sensitivity; however bimodal hotspot distributions were found in each. The lack of a trend for these variables likely derives from the percentile-based social-ecological sensitivity approach, as applying globally uniform perturbations across all basins does not change the rank-order of basin sensitivity (social or ecological) and thus yields minimal impact on our analysis. We found hotspot basins to be very consistent throughout this spatially uniform uncertainty analysis. Across the 10,000 perturbation sets, 98% of the transitional or hotspot basins were identified as so in over 50% of the perturbation sets, while 85% of the hotspot basins (exclusively) were identified as so in over 50% of the perturbation sets. Further, only 28 basins were identified as transitional or hotspot basins in over 50% of the perturbation sets but were not identified as so in our main analysis. Only 18 basins were identified as hotspot basins (exclusively) in over 50% of the perturbation sets but were not identified as so in our main analysis. We found that there was very little impact of systematic under- and/or over-estimation of input variable datasets on our transitional and hotspot basin results, and slightly greater impact on our hotspot basin results. Thus, it appears that the impact of systematic over- or under-estimation of individual variables could minorly impact our differentiation of hotspot basins from transitional basins, but does not significantly impact our differentiation of transitional basins from non-vulnerable basins.

To explore potential spatially variable uncertainty, we similarly varied the basin-level input data for all six input variables simultaneously to our vulnerability analysis. Rather than perturbing all basins by the same magnitude for each variable per perturbation set, we perturbed basins individually based on random sampling from a normal distribution. The

perturbations between basins, between variables, and between sets were uncorrelated, as the perturbation applied per basin, per variable, per set was randomly sampled from a normal distribution where we randomly determined the distribution's variance from a uniform distribution, i.e. $N(\mu = 0, \sigma \sim U(0, 20\%$ of the global σ of the variable$))$. We repeated the hotspot analysis for 10,000 perturbation sets. Supplementary Figure 9 reveals the results of our analysis of potential spatially variable uncertainty. We found that the number of identified hotspot basins decreased as the variance in perturbations applied to streamflow rates increased. These spatially-random perturbations to all other input variables showed little to no impact on hotspot basin counts. We found our hotspot basins to be very consistent throughout this spatially variable uncertainty analysis. Across the 10,000 perturbation sets, 97% of the transitional or hotspot basins were identified as so in over 50% of the perturbation sets, while 83% of the hotspot basins (exclusively) were identified as so in over 50% of the perturbation sets. Further, only 19 basins were identified as transitional or hotspot basins in over 50% of the perturbation sets but were not identified as so in our main analysis. Only 1 basin was identified as a hotspot basin in over 50% of the perturbation sets but was not identified as so in our main analysis. Thus, we similarly found that potential spatially variable uncertainty in our input data has limited impact on our derived hotspot basins.

We also considered the impact of our subjective methodological decisions on our hotspot basin results. To account for the subjective decisions central to this study, we performed the hotspot derivation process for alternative combinations of basin scale, input data where suitable data alternatives exist, and indicator aggregation methods. While we implemented HydroBASINS level 4 as our basin discretization scheme for our main analysis, we considered here the alternative scales of HydroBASINS level 3 and 5 to address the potential impact of the modifiable areal unit problem on our results[36]. There are also competing definitions of freshwater stress that vary in their consideration of withdrawals versus consumption rates. Another subjective decision we made was in regard to streamflow, as multiple gridded streamflow datasets exist though we used only one in our main analysis. Finally, we aggregated social and ecological sensitivity using the fuzzy sum operator, however arithmetic averaging is the more common approach in indicator-based studies. We thus computed hotspot basins for 24 alternative method configurations: scale (3 alternatives: HydroBASINS level 3, 4, 5), demand (2 alternatives: consumption, withdrawal), streamflow (2 alternatives: Global Streamflow Characteristics Dataset - GSCD, Gridded Runoff - GRUN), and sensitivity aggregation (2 alternatives: fuzzy sum, arithmetic mean). The frequency of individual grid cells being identified as transitional and hotspot basins through these alternative methodologies are shown in Supplementary Figure 10. Similarly to our uncertainty analysis, we found that our subjective methodological decisions had minimal effect on transitional and hotspot basins and slightly more effect on the hotspot basin results. We found the hotspot basins in our main analysis to be identified as transitional or hotspot basins in the majority of the alternative configurations. While some regions were identified as transitional basins in alternative method configurations, they were only identified as so in a small minority of these combinations. Conversely, there were a few hotspot basins in our main analysis (e.g. southwestern Australia) that were only identified as hotspot basins in a minority of the alternative configurations. This finding indicates that the methodological configuration we

selected and reported on in the main text was one of the few configurations that lead to these basins being identified as hotspots. However, these basins were still identified as transitional basins in several configurations. This analysis demonstrates one clear effect of subjective decision making on our overall hotspot results.

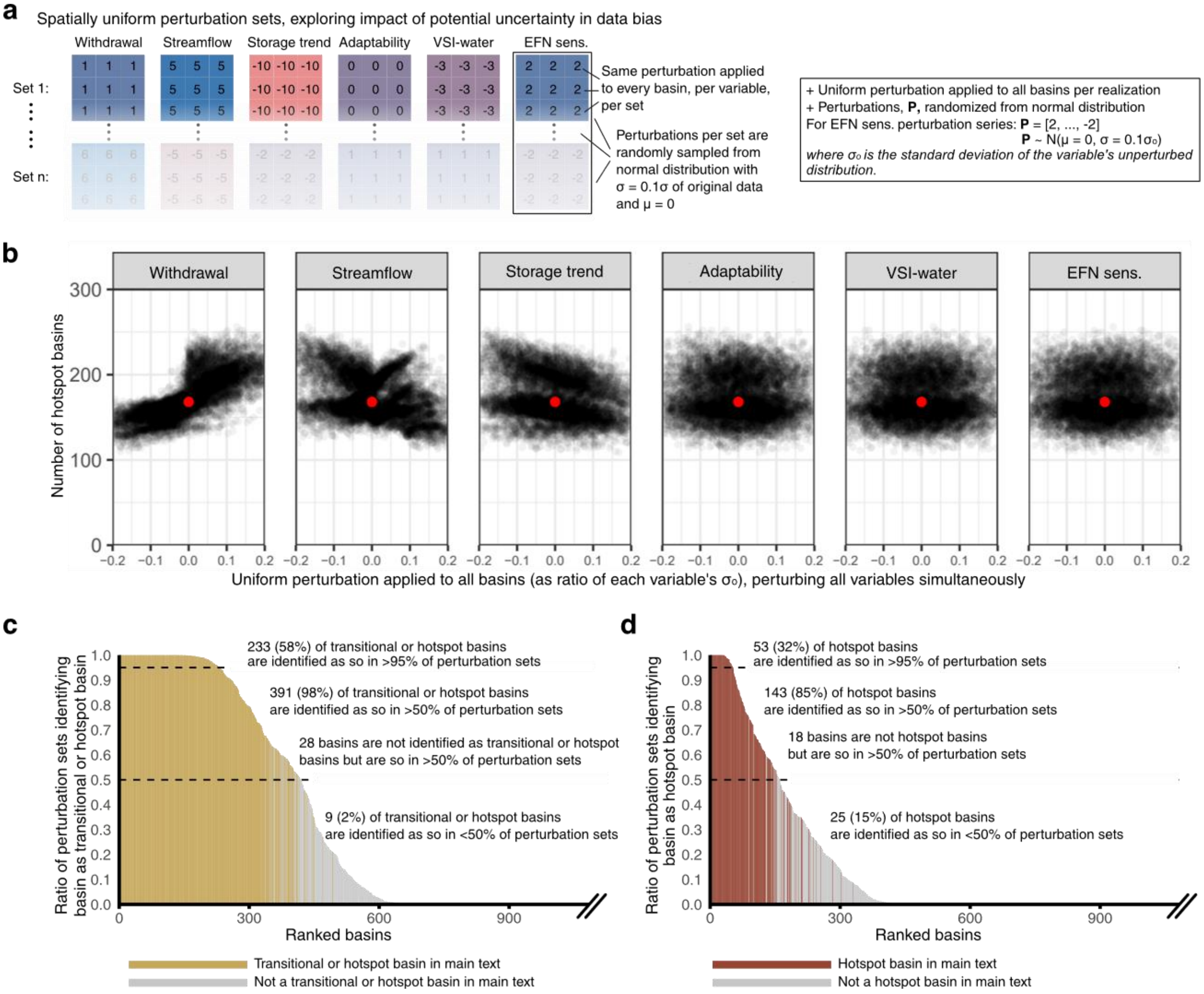

Supplementary Fig. 8: Analysis of potential spatially uniform data uncertainty. a, Schematic describing the uncertainty analysis approach. **b**, Scatterplot of hotspot basin counts from 10,000 random perturbation sets, plotted against individual perturbation magnitudes per variable. The number of hotspot basins identified in the main text is shown by the red circle in each plot. **c**, Frequency of basins being identified as either a transitional or hotspot basin over the 10,000 perturbation sets. Transitional or hotspot basins in the main text are coloured yellow. **d**, Frequency of basins being identified as a hotspot basin over the 10,000 perturbation sets. Hotspot basins in the main text are coloured red.

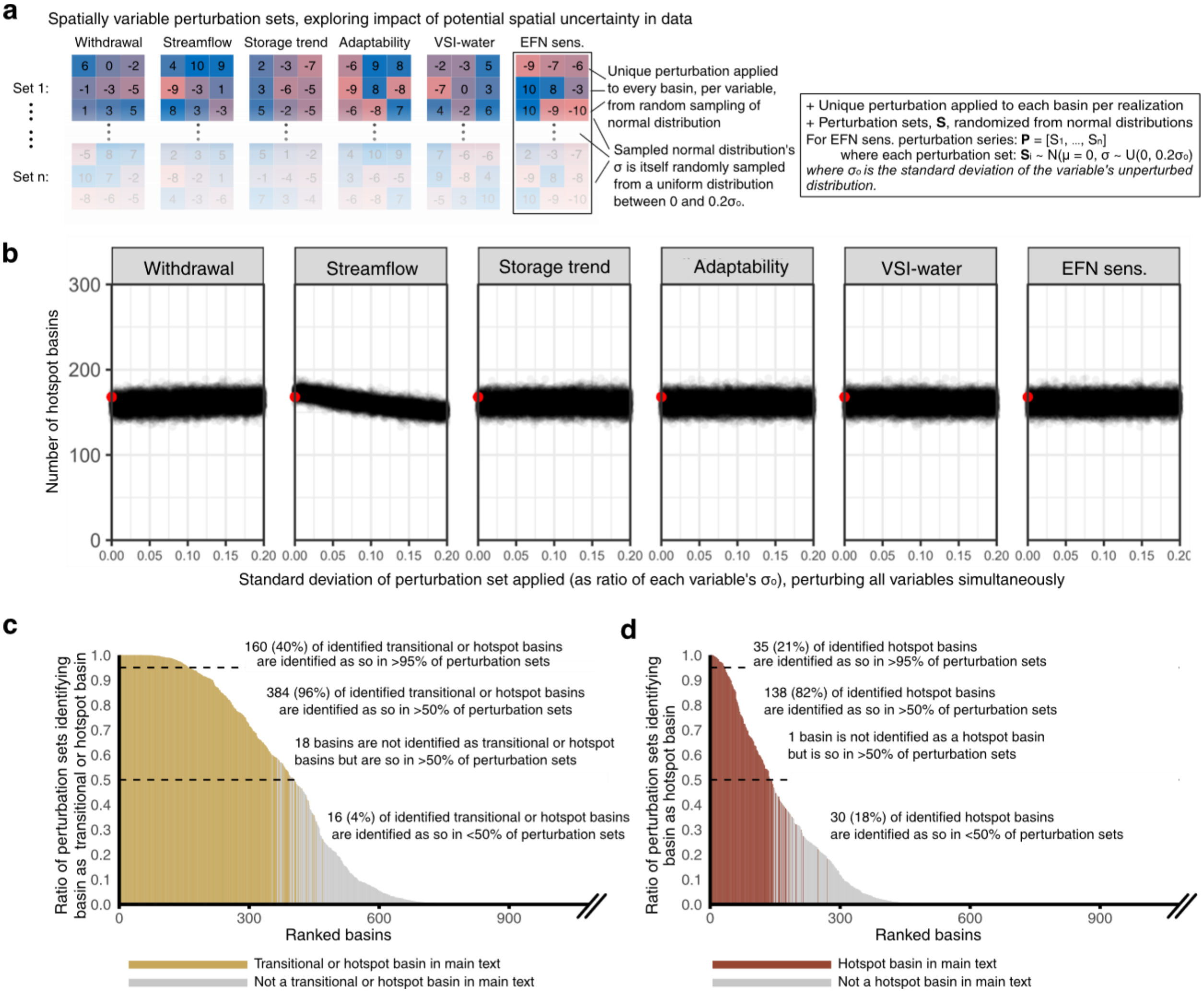

Supplementary Fig. 9: Analysis of potential spatially variable data uncertainty. a, Schematic describing the uncertainty analysis approach. **b**, Scatterplot of hotspot basin counts from 10,000 random perturbation sets, plotted against individual perturbation magnitudes per variable. The number of hotspot basins identified in the main text is shown by the red circle in each plot. **c**, Frequency of basins being identified as either a transitional or hotspot basin over the 10,000 perturbation sets. Transitional or hotspot basins in the main text are coloured yellow. **d**, Frequency of basins being identified as a hotspot basin over the 10,000 perturbation sets. Hotspot basins in the main text are coloured red.

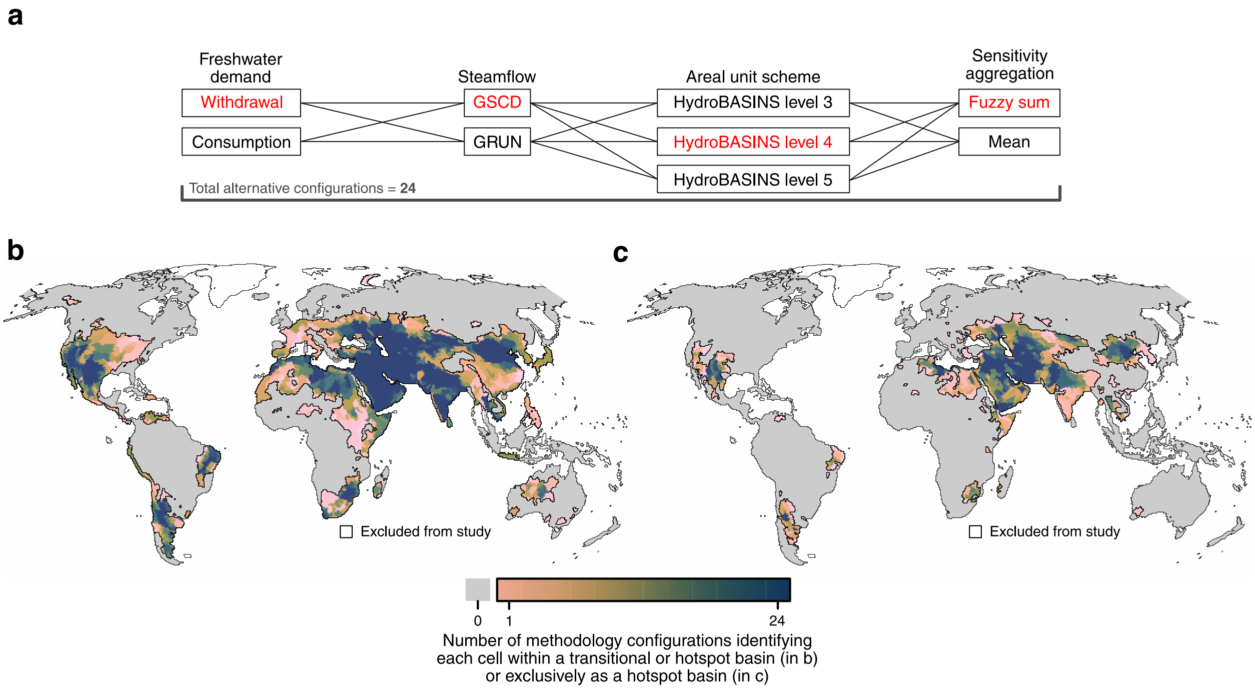

Supplementary Fig. 10: The sensitivity of hotspot basin results to subjective aspects of the study's methodology. **a**, Schematic of the various methodological configurations evaluated in this sensitivity analysis. The configuration used in the main analysis is shown by the alternatives in red text. **b**, The frequency of methodological configurations identifying each grid cell as at least a transitional basin. **c**, The frequency of methodological configurations identifying each grid cell as a hotspot.

Section 5: Supplementary results

Supplementary Table 2: Wetlands of international importance (Ramsar Sites) located in hotspot basins.

Ramsar Site No.	Site name	Latitude	Longitude	Vulnerability class
36	Miankaleh Peninsula, Gorgan Bay and Lapoo-Zaghmarz Ab-bandan	36.83	53.70	Hotspot; High vulnerability
37	Lake Parishan and Dasht-e-Arjan	29.50	52.00	Hotspot; High vulnerability
38	Lake Urmia [or Orumiyeh]	37.50	45.50	Hotspot; High vulnerability
39	Neiriz Lakes & Kamjan Marshes	29.67	53.50	Hotspot; Very high vulnerability
40	Anzali Wetland Complex	37.42	49.47	Hotspot; High vulnerability
41	Shadegan Marshes & mudflats of Khor-al Amaya & Khor Musa	30.50	48.75	Hotspot; High vulnerability
42	Hamun-e-Saberi & Hamun-e-Helmand	31.24	61.25	Hotspot; Very high vulnerability
43	Lake Kobi	36.95	45.50	Hotspot; High vulnerability
44	Hamun-e-Puzak, south end	31.33	61.75	Hotspot; High vulnerability
45	Shurgol, Yadegarlu & Dorgeh Sangi Lakes	37.00	45.50	Hotspot; High vulnerability
46	Bujagh National Park	37.42	49.48	Hotspot; High vulnerability
48	Lake Gori	37.92	46.70	Hotspot; High vulnerability
49	Alagol, Ulmagol and Ajigol Lakes	37.35	54.58	Hotspot; Very high vulnerability
50	Khuran Straits	26.75	55.67	Hotspot; Very high vulnerability
51	Deltas of Rud-e-Shur, Rud-e-Shirin and Rud-e-Minab	27.09	56.75	Hotspot; High vulnerability
52	Deltas of Rud-e-Gaz and Rud-e-Hara	26.67	57.33	Hotspot; High vulnerability
53	Gavkhouni Lake and marshes of the lower Zaindeh Rud	32.33	52.78	Hotspot; Very high vulnerability
99	Kinjhar Lake	24.93	68.05	Hotspot; Very high vulnerability
108	Lakes of the lower Turgay and Irgiz	48.70	62.18	Hotspot; High vulnerability
111	Volga Delta	45.90	48.78	Hotspot; Very high vulnerability
135	Azraq Oasis	31.82	36.80	Hotspot; Very high vulnerability

230	Keoladeo National Park	27.22	77.53	Hotspot; High vulnerability
343	Blesbokspruit	-26.31	28.50	Hotspot; High vulnerability
347	Ash Meadows National Wildlife Refuge	36.42	-116.33	Hotspot; High vulnerability
380	Koshi Tappu	26.65	86.98	Hotspot; High vulnerability
409	Xuan Thuy Natural Wetland Reserve	20.17	106.33	Hotspot; High vulnerability
461	Wular Lake	34.27	74.55	Hotspot; High vulnerability
462	Harike Lake	31.22	75.20	Hotspot; High vulnerability
464	Sambhar Lake	27.00	75.00	Hotspot; High vulnerability
483	Toolibin Lake	-32.92	117.61	Hotspot; High vulnerability
560	Sundarbans Reserved Forest	22.03	89.52	Hotspot; High vulnerability
561	Mühlenberger Loch	53.53	9.80	Hotspot; High vulnerability
620	Lake Sevan	40.26	45.36	Hotspot; High vulnerability
621	Lake Arpi	41.06	43.64	Hotspot; High vulnerability
672	Veselovskoye Reservoir	46.92	41.03	Hotspot; Very high vulnerability
673	Lake Manych-Gudilo	44.60	42.83	Hotspot; Very high vulnerability
734	Área de Protección de Flora y Fauna Cuatrociénegas	26.85	-102.13	Hotspot; High vulnerability
868	Hula Nature Reserve	33.07	35.58	Hotspot; Very high vulnerability
887	Ndumo Game Reserve	-26.88	32.27	Hotspot; High vulnerability
920	Hawar Islands	25.67	50.83	Hotspot; High vulnerability
921	Tubli Bay	26.18	50.57	Hotspot; High vulnerability
952	Nylsvley Nature Reserve	-24.65	28.70	Hotspot; High vulnerability
998	Koh Kapik and Associated Islets	11.47	103.07	Hotspot; Very high vulnerability
1006	Govater Bay and Hur-e-Bahu	25.17	61.50	Hotspot; High vulnerability
1012	Lagunas de Guanacache, Desaguadero y del Bebedero	-33.00	-67.60	Hotspot; Very high vulnerability
1015	Sheedvar Island	26.80	53.40	Hotspot; Very high vulnerability

1026	Ain Elshakika	32.77	21.35	Hotspot; Very high vulnerability
1027	Ain Elzarga	32.78	22.35	Hotspot; Very high vulnerability
1054	Chott Merrouane et Oued Khrouf	33.89	6.18	Hotspot; High vulnerability
1066	Jiwani Coastal Wetland	25.08	61.80	Hotspot; High vulnerability
1067	Jubho Lagoon	24.33	68.67	Hotspot; Very high vulnerability
1069	Nurri Lagoon	24.50	68.78	Hotspot; Very high vulnerability
1075	Agh-Ghol	40.02	47.63	Hotspot; High vulnerability
1076	Ghizil-Agaj	39.12	48.98	Hotspot; High vulnerability
1083	Kayrakum Reservoir	40.33	70.17	Hotspot; High vulnerability
1108	Lake Dengizkul	39.12	64.17	Hotspot; High vulnerability
1109	Gomishan Lagoon	37.20	53.98	Hotspot; Very high vulnerability
1110	Verloren Valei Nature Reserve	-25.31	30.11	Hotspot; High vulnerability
1148	Eerduosi National Nature Reserve	39.80	109.58	Hotspot; Very high vulnerability
1160	Kanjli	31.42	75.37	Hotspot; High vulnerability
1161	Ropar	31.02	76.50	Hotspot; High vulnerability
1205	Bhitarkanika Mangroves	20.65	86.90	Hotspot; High vulnerability
1206	Bhoj Wetland	23.23	77.33	Hotspot; High vulnerability
1208	East Calcutta Wetlands	22.45	88.45	Hotspot; High vulnerability
1211	Pong Dam Lake	32.02	76.08	Hotspot; High vulnerability
1213	Tsomoriri	32.90	78.30	Hotspot; High vulnerability
1285	Runn of Kutch	24.38	70.08	Hotspot; Very high vulnerability
1296	Chott Melghir	34.25	6.51	Hotspot; High vulnerability
1313	Beeshazar and Associated Lakes	27.62	84.43	Hotspot; High vulnerability
1314	Ghodaghodi Lake Area	28.68	80.95	Hotspot; High vulnerability
1315	Jagadishpur Reservoir	27.58	83.08	Hotspot; High vulnerability

1326	Playa Tortuguera Rancho Nuevo	23.23	-97.77	Hotspot; High vulnerability
1362	Laguna Madre	24.73	-97.58	Hotspot; High vulnerability
1378	Lake Ganga and its surrounding wetlands	45.25	114.00	Hotspot; High vulnerability
1399	Dnipro-Oril Floodplains	48.53	34.75	Hotspot; High vulnerability
1417	Chott Sidi Slimane	33.29	6.05	Hotspot; High vulnerability
1439	Mapangyong Cuo	30.69	81.39	Hotspot; High vulnerability
1441	Shuangtai Estuary	40.91	121.76	Hotspot; High vulnerability
1569	Chandertal Wetland	32.48	77.60	Hotspot; High vulnerability
1570	Hokera Wetland	34.08	74.70	Hotspot; High vulnerability
1571	Renuka Wetland	31.62	77.45	Hotspot; High vulnerability
1573	Surinsar-Mansar Lakes	32.75	75.20	Hotspot; High vulnerability
1574	Upper Ganga River	28.55	78.20	Hotspot; High vulnerability
1588	Chatyr Kul	40.62	75.30	Hotspot; High vulnerability
1687	Makuleke Wetlands	-22.39	31.20	Hotspot; High vulnerability
1692	Gokyo and associated lakes	27.95	86.68	Hotspot; High vulnerability
1693	Gosaikunda and Associated Lakes	28.08	85.43	Hotspot; High vulnerability
1694	Phoksundo Lake	29.20	82.95	Hotspot; High vulnerability
1695	Rara Lake	29.50	82.08	Hotspot; High vulnerability
1697	Bahiret el Bibane	33.25	11.22	Hotspot; Very high vulnerability
1699	Chott El Jerid	33.70	8.40	Hotspot; High vulnerability
1700	Djerba Bin El Ouedian	33.67	10.92	Hotspot; Very high vulnerability
1701	Djerba Guellala	33.70	10.73	Hotspot; Very high vulnerability
1702	Djerba Ras Rmel	33.87	10.90	Hotspot; Very high vulnerability
1714	Zones humides oasiennes de Kebili	33.50	8.92	Hotspot; High vulnerability
1718	Hawizeh Marsh	31.42	47.63	Hotspot; Very high vulnerability

1762	Laguna de Babícora	29.33	-107.83	Hotspot; High vulnerability
1769	Río Sabinas	27.88	-101.15	Hotspot; High vulnerability
1822	Sistema de Humedales Remanentes del Delta del Río Colorado	32.32	-115.25	Hotspot; High vulnerability
1841	Aydar-Arnasay Lakes system	40.78	67.77	Hotspot; High vulnerability
1850	Mai Pokhari	27.00	87.92	Hotspot; High vulnerability
1855	Turkmenbashy Bay	39.78	53.35	Hotspot; High vulnerability
1863	Kulykol-Taldykol Lake System	51.38	61.87	Hotspot; High vulnerability
1872	Naurzum Lake System	51.49	64.30	Hotspot; High vulnerability
1873	Zharsor-Urkash Lake System	51.32	62.73	Hotspot; High vulnerability
1890	Lake Kuyucuk	40.75	43.45	Hotspot; High vulnerability
1892	Alakol-Sasykkol Lakes System	46.27	81.53	Hotspot; High vulnerability
1917	Roswell Artesian Wetlands	33.45	-104.38	Hotspot; High vulnerability
1939	Choghakhor Wetland	31.92	50.90	Hotspot; Very high vulnerability
1940	Kanibarazan Wetland	37.00	45.77	Hotspot; High vulnerability
1943	Son-Kol Lake	41.83	75.12	Hotspot; High vulnerability
1981	Baño de San Ignacio	24.87	-99.34	Hotspot; High vulnerability
1989	Khor Virap Marsh	39.89	44.57	Hotspot; High vulnerability
2005	Chott Elguetar	34.29	8.91	Hotspot; High vulnerability
2007	Marais d'eau douce Garaet Douza	34.47	8.48	Hotspot; High vulnerability
2008	Golfe de Boughrara	33.47	10.75	Hotspot; Very high vulnerability
2009	Les Gorges de Thelja	34.15	8.28	Hotspot; High vulnerability
2011	Oued Dekouk	32.34	10.61	Hotspot; Very high vulnerability
2047	Río San Pedro - Meoqui	28.28	-105.44	Hotspot; High vulnerability
2070	Humedales de Península Valdés	-42.46	-64.30	Hotspot; Very high vulnerability
2083	Lesser Aral Sea and Delta of the Syrdarya River	46.35	61.00	Hotspot; High vulnerability

2088	Mui Ca Mau National Park	8.68	104.79	Hotspot; Very high vulnerability
2100	Complexe des zones humides de Sebkhet Oum Ez-Zessar et Sebkhet El Grine	33.65	10.52	Hotspot; Very high vulnerability
2123	Van Eck Dam	-26.77	31.92	Hotspot; High vulnerability
2187	Shandong Yellow River Delta Wetland	37.77	119.09	Hotspot; Very high vulnerability
2201	Manantiales Geotermales de Julimes	28.41	-105.43	Hotspot; High vulnerability
2203	Con Dao National Park	8.71	106.64	Hotspot; Very high vulnerability
2206	Laguna La Juanota	26.49	-106.47	Hotspot; High vulnerability
2224	Complexe des lacs Ambondro et Sirave (CLAS)	-20.91	43.94	Hotspot; High vulnerability
2228	U Minh Thuong National Park	9.59	105.10	Hotspot; Very high vulnerability
2239	Mubarak Al-Kabeer Reserve	29.90	48.13	Hotspot; High vulnerability
2240	Sawa Lake	31.31	45.01	Hotspot; Very high vulnerability
2241	Central Marshes	31.18	46.98	Hotspot; Very high vulnerability
2242	Hammar Marsh	30.81	47.02	Hotspot; Very high vulnerability
2257	Lake Cluster of Pokhara Valley	28.21	83.98	Hotspot; High vulnerability
2282	Archipelago Velyki and Mali Kuchugury	47.56	35.20	Hotspot; High vulnerability
2294	Fifa Nature Reserve	30.96	35.44	Hotspot; Very high vulnerability
2302	Mangroves de Tsiribihina	-19.74	44.46	Hotspot; High vulnerability
2360	Van Long Wetland Nature Reserve	20.39	105.85	Hotspot; High vulnerability
2369	Zarivar	35.54	46.13	Hotspot; High vulnerability
2370	Sundarban Wetland	21.77	88.71	Hotspot; High vulnerability
2407	Nangal Wildlife Sanctuary	31.40	76.37	Hotspot; High vulnerability
2408	Beas Conservation Reserve	31.39	75.19	Hotspot; High vulnerability
2409	Sandi Bird Sanctuary	27.31	79.97	Hotspot; High vulnerability
2411	Sarsai Nawar Jheel	26.97	79.25	Hotspot; High vulnerability
2412	Nawabganj Bird Sanctuary	26.61	80.65	Hotspot; High vulnerability

2413	Saman Bird Sanctuary	27.02	79.18	Hotspot; High vulnerability
2414	Keshopur-Miani Community Reserve	32.09	75.39	Hotspot; High vulnerability
2415	Samaspur Bird Sanctuary	26.00	81.39	Hotspot; High vulnerability
2416	Parvati Arga Bird Sanctuary	26.94	82.16	Hotspot; High vulnerability
2425	Tianjin Beidagang Wetlands	38.79	117.36	Hotspot; Very high vulnerability
2433	Tudakul and Kuymazar Water Reservoirs	39.85	64.83	Hotspot; High vulnerability
2434	Bugdasheni Lake	41.20	43.68	Hotspot; High vulnerability
2435	Madatapa Lake	41.18	43.78	Hotspot; High vulnerability
2436	Kabartal Wetland	25.62	86.14	Hotspot; High vulnerability
2437	Asan Conservation Reserve	30.43	77.68	Hotspot; High vulnerability
2440	Sur Sarovar	27.25	77.84	Hotspot; High vulnerability

References

1. Xu, L., Mao, F., Famiglietti, J. S., Pomeroy, J. W. & Pahl-Wostl, C. Conceptualizing Cascading Effects of Resilience in Human–Water Systems. in *Multisystemic Resilience: Adaptation and Transformation in Contexts of Change* 744–767 (Oxford University Press, 2021).
2. Falkenmark, M. & Wang-Erlandsson, L. A water-function-based framework for understanding and governing water resilience in the Anthropocene. *One Earth* **4**, 213–225 (2021).
3. Gleeson, T. *et al.* The Water Planetary Boundary: Interrogation and Revision. *One Earth* **2**, 223–234 (2020).
4. Varis, O., Taka, M. & Kummu, M. The Planet's Stressed River Basins: Too Much Pressure or Too Little Adaptive Capacity? *Earth's Future* **7**, 1118–1135 (2019).
5. Whittaker, R. J. *et al.* Conservation Biogeography: assessment and prospect. *Diversity and Distributions* **11**, 3–23 (2005).
6. Lei, Y., Wang, J., Yue, Y., Zhou, H. & Yin, W. Rethinking the relationships of vulnerability, resilience, and adaptation from a disaster risk perspective. *Nat Hazards* **70**, 609–627 (2014).
7. Turner, B. L. *et al.* A framework for vulnerability analysis in sustainability science. *Proceedings of the National Academy of Sciences of the United States of America* **100**, 8074–8079 (2003).
8. Mao, F. *et al.* HESS Opinions: A conceptual framework for assessing socio-hydrological resilience under change. *Hydrology and Earth System Sciences* **21**, 3655–3670 (2017).
9. Rodell, M. *et al.* Emerging trends in global freshwater availability. *Nature* **557**, 651–659 (2018).
10. Biggs, R. *et al.* Toward Principles for Enhancing the Resilience of Ecosystem Services. *Annu. Rev. Environ. Resour.* **37**, 421–448 (2012).
11. Dai, A. Historical and Future Changes in Streamflow and Continental Runoff. in *Terrestrial Water Cycle and Climate Change* 17–37 (2016).
12. Huang, Z. *et al.* Reconstruction of global gridded monthly sectoral water withdrawals for 1971–2010 and analysis of their spatiotemporal patterns. *Hydrology and Earth System Sciences* **22**, 2117–2133 (2018).
13. Huang, Z. *et al.* Global gridded monthly sectoral water use dataset for 1972-2010: v2. *Zenodo* https://doi.org/10.5281/zenodo.1209296 (2018).
14. Beck, H. E. *et al.* Global patterns in base flow index and recession based on streamflow observations from 3394 catchments. *Water Resources Research* **49**, 7843–7863 (2013).
15. Beck, H. E., Roo, A. de & Dijk, A. I. J. M. van. Global Maps of Streamflow Characteristics Based on Observations from Several Thousand Catchments. *Journal of Hydrometeorology* **16**, 1478–1501 (2015).
16. Beck et al. GSCD: Global Streamflow Characteristics Dataset version 2.0. *GloH2O* http://www.gloh2o.org/gscd/ (2021).
17. Ghiggi, G., Humphrey, V., Seneviratne, S. I. & Gudmundsson, L. GRUN: an observation-based global gridded runoff dataset from 1902 to 2014. *Earth System Science Data* **11**, 1655–1674 (2019).
18. Ghiggi, G., Humphrey, V., Seneviratne, S. I. & Gudmundsson, L. GRUN: Global Runoff Reconstruction. *Figshare* https://doi.org/10.6084/m9.figshare.9228176 (2019).

19. Seddon, A. W. R., Macias-Fauria, M., Long, P. R., Benz, D. & Willis, K. J. Sensitivity of global terrestrial ecosystems to climate variability. *Nature* **531**, 229–232 (2016).
20. Seddon, A. W. R., Macias-Fauria, M., Long, P. R., Benz, D. & Willis, K. J. Sensitivity of global terrestrial ecosystems to climate variability: data and R code. *Oxford University Research Archive* https://doi.org/10.5287/bodleian:VY2PeyGX4 (2016).
21. de Graaf, I. E. M., Gleeson, T., (Rens) van Beek, L. P. H., Sutanudjaja, E. H. & Bierkens, M. F. P. Environmental flow limits to global groundwater pumping. *Nature* **574**, 90–94 (2019).
22. de Graaf, I. E. M., Gleeson, T., (Rens) van Beek, L. P. H., Sutanudjaja, E. H. & Bierkens, M. F. P. Environmental flow limits to global groundwater pumping. *Scholars Portal Dataverse* https://doi.org/10.5683/SP2/D7I7CC (2019).
23. Varis, O., Taka, M. & Kummu, M. Data from: The planet's stressed river basins: too much pressure or too little adaptive capacity? *Dryad* https://doi.org/10.5061/dryad.h2v2398 (2019).
24. Center for International Earth Science Information Network - CIESIN - Columbia University. Gridded Population of the World, Version 4 (GPWv4): Population Density Adjusted to Match 2015 Revision UN WPP Country Totals, Revision 11. *NASA SEDAC* https://doi.org/10.7927/H4PN93PB (2018).
25. Doxsey-Whitfield, E. *et al.* Taking Advantage of the Improved Availability of Census Data: A First Look at the Gridded Population of the World, Version 4. *Papers in Applied Geography* **1**, 226–234 (2015).
26. Kummu, M., Heino, M., Taka, M., Varis, O. & Viviroli, D. Climate change risks pushing one-third of global food production outside the safe climatic space. *One Earth* **4**, 720–729 (2021).
27. International Food Policy Research Institute. Global Spatially-Disaggregated Crop Production Statistics Data for 2010 Version 2.0. *Harvard Dataverse* https://doi.org/10.7910/DVN/PRFF8V (2019).
28. Kummu, M., Taka, M. & Guillaume, J. H. A. Gridded global datasets for Gross Domestic Product and Human Development Index over 1990-2015. *Scientific Data* **5**, 1–15 (2018).
29. Kummu, M., Taka, M. & Guillaume, J. H. A. Data from: Gridded global datasets for Gross Domestic Product and Human Development Index over 1990-2015. *Dryad* https://doi.org/10.5061/dryad.dk1j0 (2019).
30. International Union for Conservation of Nature - IUCN, and Center for International Earth Science Information Network - CIESIN - Columbia University. Gridded Species Distribution: Global Amphibian Richness Grids, 2015 Release. *NASA SEDAC* https://doi.org/10.7927/H4RR1W66 (2015).
31. Tisseuil, C. *et al.* Global diversity patterns and cross-taxa convergence in freshwater systems. *J Anim Ecol* **82**, 365–376 (2013).
32. Ramsar Convention Secretariat. Ramsar Sites Information Service. https://rsis.ramsar.org/ (2021).
33. UNEP-DHI Centre on Water and Environment. IWRM Data Portal. http://iwrmdataportal.unepdhi.org/ (2021).

34. Lehner, B. & Grill, G. Global river hydrography and network routing: baseline data and new approaches to study the world's large river systems. *Hydrological Processes* **27**, 2171–2186 (2013).
35. Lehner, B. & Grill, G. HydroBASINS Version 1.c. *World Wildlife Foundation HydroSHEDS* https://www.hydrosheds.org/page/hydrobasins (2014).
36. Dark, S. J. & Bram, D. The modifiable areal unit problem (MAUP) in physical geography. *Progress in Physical Geography: Earth and Environment* **31**, 471–479 (2007).
